# A New Mechanism for Ginsenoside Rb1 to Promote Glucose Uptake, Regulating Riboflavin Metabolism and Redox Homeostasis

**DOI:** 10.3390/metabo12111011

**Published:** 2022-10-23

**Authors:** Yihan Liu, Yuchan Deng, Fengyu Wang, Xiaoyi Liu, Jiaqi Wang, Jian Xiao, Cunli Zhang, Qiang Zhang

**Affiliations:** 1 Shaanxi Key Laboratory of Natural Products & Chemical Biology, College of Chemistry & Pharmacy, Northwest A&F University, Xianyang 712100, China; 2 Shaanxi Key Laboratory of Phytochemistry, College of Chemistry and Chemical Engineering, Baoji University of Arts and Sciences, Baoji 721013, China

**Keywords:** *Panax quinquefolius* L., bioguided isolation, diabetes, metabolomics, riboflavin, redox

## Abstract

Glucose absorption promoters perform insulin mimic functions to enhance blood glucose transport to skeletal muscle cells and accelerate glucose consumption, thereby reducing blood glucose levels. In our screening exploration of food ingredients for improving glucose transportation and metabolism, we found that the saponins in American ginseng (*Panax* *quinquefolius* L.) showed potential activity to promote glucose uptake, which can be used for stabilizing levels of postprandial blood glucose. The aim of this study was to identify key components of American ginseng with glucose uptake-promoting activity and to elucidate their metabolic regulatory mechanisms. Bio-guided isolation using zebrafish larvae and 2-NBDG indicator identified ginsenoside Rb1 (GRb1) as the most potential promotor of glucose uptake. Using UPLC-QTOF-MS/MS combined with RT-qPCR and phenotypic verification, we found that riboflavin metabolism is the hinge for GRb1-mediated facilitation of glucose transport. GRb1-induced restoration of redox homeostasis was mediated by targeting riboflavin transporters (SLC52A1 and SLC52A3) and riboflavin kinase (RFK).

## 1. Introduction

Diabetes induced by glucose metabolism disorder is prevalent in older people and has now become a global medical and social burden [1]. Chronic hyperglycemia plays a crucial role in the development and progression of diabetes [2]. Long-term chronic hyperglycemia induces insulin resistance and oxidative stress in the body, triggering damage to various tissues and organs such as the liver, pancreas, muscle, kidney, heart, retina and cardiovascular and cerebrovascular tissues, further forming chronic functional lesions and leading to the development of various diabetic complications [3]. Impaired glucose tolerance (IGT) is an abnormal state of glucose metabolism in the transition from normoglycemia to diabetes, mainly manifested as elevated postprandial glucose. It is now believed that almost all people with diabetes go through the IGT stage, so it is also known as pre-diabetes. According to statistics, nearly half (48.1%) of the global IGT population is under the age of 50. This means that, once they develop diabetes, they will have a long lifetime of carrying the disease and will also need to manage the risk of complications from diabetes. Thus, it is more meaningful to delay diabetes development by reasonable diet intervention than medical treatment during pre-diabetes.

Herbs have unique efficacy in promoting health, preventing and assisting in treating chronic diseases [4]. Two plants of *Araliaceae* family, *Panax quinquefolius* L. (American) and *Panax ginseng* C.A. Meyer (Asian), are the two major ginseng plants recognized worldwide for their medicinal uses. The roots of ginseng herbs have been used for the treatment and prevention of various diseases for more than 2000 years [5], and their stems, leaves, flowers and fruits are not only critical medicinal resources but also used as functional ingredients in foods and cosmetics [6]. In addition, ginseng (*P. ginseng*) and American ginseng (*P. quinquefolius*) are also certified as medicinal and food substances in China. The most typical active ingredient in ginseng plants is triterpene saponin, also known as ginsenoside. Modern pharmacological studies have shown that ginsenosides have shown various activities, such as antitumor, cardiovascular system protection, neuroprotection, immune function improvement, and antidiabetic effects [7,8,9].

Glucose metabolism disorders are highly related to metabolism [10]. Metabolomics provides a high-throughput method to investigate the small molecular response to biological or treated processes inside an organism. Differential expression of metabolites’ response to treatment can reflect the main regulatory points of drugs on metabolism in vivo [11]. Furthermore, metabolomics is closely related to phenotyping. It has been rapidly developing and penetrating many fields concerning human health care, such as disease diagnosis, pharmaceutical development, nutritional food science, toxicology, environmental science, botany, etc.

In this study, we employed zebrafish larvae as an exploratory model. The most promising component to regulate glucose uptake was obtained from American ginseng by activity tracing, and its metabolic regulatory mechanism was explored in a diabetes model using a metabolomics approach. This will provide a basis for rational use of dietary resources to prevent and control early diabetes.

## 2. Materials and Methods

### 2.1. Materials

The herb was purchased from the herb market (Guo Xin Pharmacy, Jiangsu, China) and identified as the root of *Panax quinquefolius* L. by Prof. Cunli Zhang of Northwest Agriculture and Forestry University of Science and Technology. We purchased 2-Deoxy-2-[(7-nitro-2,1,3-benzoxadiazol-4-yl) amino]-D-glucose (2-NBDG) from Shanghai Dibai Biotechnology Co., Ltd. (Shanghai, China). N-Phenylthiourea (PTU), tricaine, alloxan, 2,7-Dichlorodihydrofluorescein diacetate (DCFH-DA), acridine orange (AO), riboflavin, catalase (CAT) activity assay kit and malondialdehyde (MDA) content assay kit were purchased from Beijing Solarbio Science & Technology Co., Ltd. (Beijing, China).

### 2.2. Zebrafish Maintenance and Embryo Collection

Wild-type AB line adult zebrafish (*Danio rerio*) were housed in rearing tanks under the following conditions: 28.0 °C, alternating light (14 h) and dark (10 h), and the fish were fed three times a day. Zebrafish were placed in a constant temperature incubator for spawning. Male and female zebrafish were placed in spawning boxes at a ratio of 1/2 and isolated with a partition overnight. The next morning, the spacer was removed, the light was given, and spawning was triggered. Collected eggs were incubated in E3 water (5 mM NaCl, 0.17 mM KCl, 0.33 mM CaCl_2_ and 0.33 mM MgSO_4_) supplemented with 0.2 mM PTU (a decolorizing agent that increases larval transparency). The incubation solution was changed daily.

### 2.3. Toxicity Evaluation on Zebrafish Larvae

The 72 hpf (hour post fertilization) larvae were divided into blank and test groups. Thirty larvae were in each group. The blank group was exposed to E3 water, and the test groups were exposed to E3 water containing the test drugs. The larvae were continuously exposed up to 120 hpf. The morphology and motility of the larvae were observed under a microscope every 12 h during the exposure period. The toxicity of the drug to the larvae was evaluated.

### 2.4. Glucose Uptake Assay on Zebrafish Larvae

We used the method described in [12] for this operation [12]. Four 72 hpf larvae were placed in one well of a 96-well plate, to which was added 200 μL of E3 water, with or without the test compound. The larvae were first immersed in the test compound for 1 h, and then incubated with 2-NBDG (0.6 mM) in E3 water for 3 h. After incubation, the larvae were rinsed with E3 water and given 0.04% tricaine anesthesia. The larvae were gently laid on glass slides and imaged using an SMZ25 Fluorescence stereomicroscope (Nikon Corp, Tokyo, Japan). The 2-NBDG uptake by larvae was quantified using Image J 1.48 (National Institutes of Health).

### 2.5. Bioguided Isolation

**Figure 1:**
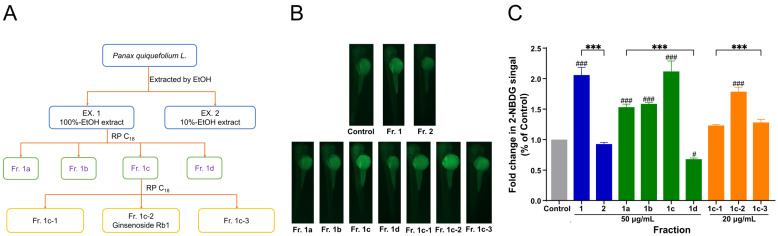
Bioguided separation of the active ingredients in American ginseng. (**A**) Scheme of bioguided fractionation. (**B**,**C**) Fluorescence microscopy images of zebrafish larvae after treatment with different fractions and the fold change in absorption of 2-NBDG quantified by Image J. Blue, green and orange are three different batches. Results are expressed as mean ± SEM. ^###^ *p* < 0.001, ^#^ *p* < 0.05 vs. control. *** *p* < 0.001.

Powdered American ginseng root (20 g) was added to 200 mL of EtOH and extracted at reflux for 2 h. After filtration, the residue was extracted with 200 mL of 10% ethanol at reflux for 2 h. The two extracts were concentrated to obtain the crude extracts EX1 (1.708 g) and EX2 (5.528 g), respectively. The two extracts were separated on a 20AP liquid chromatography system (Shimadzu, Japan) equipped with a Thermo Hypersil GOLD C_18_ column (150 mm × 50 mm, 5 μm), which was eluted by gradient MeOH from 15% to 100% for 45 min, and 100% for a further 20 min. The separated fractions Figure 1A–C were collected according to the time window of 15 min. Each fraction was diluted with E3 water and tested for toxicity and glucose uptake promoting activity using the operation methods described in Section 2.3 and Section 2.4. The 2-NBDG fluorescence intensity indicated that fraction Figure 1C was more effective in promoting glucose uptake. Fraction Figure 1C was then further purified on a YMC-Pack ODS-A column (250 mm × 10 mm, 5 μm) using 65% MeOH to yield GRb1 (123 mg). The chemical structure of the compound was identified by ^1^H, ^13^C-NMR and HR ESI-MS.

Ginsenoside Rb1. Physical state and appearance: white, amorphous powder. ^1^H NMR (400 MHz, MeOD) *δ* 5.14 (t, *J* = 6.8 Hz, 1H), 4.67 (d, *J* = 7.7 Hz, 1H), 4.59 (d, *J* = 6.9 Hz, 1H), 4.44 (d, *J* = 6.7 Hz, 1H), 4.35 (d, *J* = 7.7 Hz, 1H), 4.06 (d, *J* = 11.5 Hz, 1H), 3.89–3.80 (m, 4H), 3.79–3.68 (m, 2H), 3.67 (d, *J* = 4.3 Hz, 1H), 3.63 (d, *J* = 5.5 Hz, 1H), 3.58 (m, 3H), 3.43 (m, 1H), 3.37–3.33 (m, 4H), 3.27 (m, 4H), 3.249–3.165 (m, 6H), 3.13 (d, *J* = 7.4 Hz, 1H), 2.30 (q, *J* = 9.9 Hz, 1H), 2.14–1.91 (m, 4H), 1.88–1.71 (m, 6H), 1.69 (s, 3H), 1.63 (s, 3H), 1.57 (t, *J* = 11.6 Hz, 4H), 1.51–1.41 (m, 2H), 1.37 (s, 3H), 1.26 (m, 3H), 1.07 (s, 3H), 1.01 (s, 3H), 0.93 (s, 6H), 0.86 (s, 3H); ^13^C NMR (100 MHz, MeOD) *δ* 132.24, 126.02, 105.40, 104.99, 104.44, 98.08, 91.36, 84.96, 80.98, 78.52, 78.48, 78.35, 77.92, 77.92, 77.89, 77.73, 76.79, 76.30, 75.28, 75.13, 71.91, 71.69, 71.65, 71.56, 71.49, 70.22, 63.11, 62.83, 62.80, 57.54, 52.87, 52.40, 51.09, 49.26, 40.97, 40.59, 40.27, 37.91, 36.77, 35.84, 31.49, 30.81, 28.42, 27.24, 25.96, 23.87, 22.46, 19.24, 18.03, 17.39, 16.73, 16.70, 16.32. HR ESI-MS *m*/*z* Full MS [M + Na]^+^, calcd for C_54_H_92_O_23_, 1131.5922, found 1131.5905 (error 1.5 ppm).

### 2.6. Sample Preparation for LC-MS/MS Metabolism Analysis

Hyperglycemia can be induced conveniently by exposure to high glucose solution [13]. Zebrafish larvae (72 dpf) were randomly split into four groups, including control (G1), diabetes model (G2), GRb1 low dose (G3) and GRb1 high dose (G4) groups. Eight biological replicates (100 larvae per replicate) were used for each group. The control group was only exposed to E3 water. The model group was exposed to 4% glucose in E3 water. The two GRb1-treated groups (G3 and G4) were exposed to E3 water containing 4% glucose and GRb1 (100 µg/mL or 200 µg/mL). The culture solution was changed daily. After 2 days of exposure, larvae were washed with fresh E3 water three times. The larvae were quickly frozen in liquid N_2_ and kept in a −80 °C refrigerator for LC-MS/MS analysis after being collected.

### 2.7. UPLC-QTOF-MS/MS Analysis

The frozen zebrafish larvae were thawed for 5 min in an ice bath. To each larval sample was added 300 μL methanol, and this was ground in an ice bath to homogenize the larval tissue well. The supernatant was obtained after being centrifugated at 4 °C for 10 min at 8000× *g*. The supernatants were filtered through 0.22 µm membranes and analyzed using a UPLC-QTOF-ESIMS system (AB SCIEX 5600+, AB Sciex Pte. Ltd., Framingham, MA, USA) equipped with a UPLC column (Shim-pack XR-ODS, 2.0 mm × 100 mm, 1.7 µm). The injection volume was 5 μL. Solvent A was water (0.1% acetic acid, *v*/*v*) and solvent B was acetonitrile (0.1% acetic acid, *v*/*v*). The flow rate was controlled at 0.3 mL/min. The column was eluted with gradient solvent B from 10% to 100% B for 15 min, and then 100% B for a further 3 min. Mass spectra were acquired by the DIA SWATH method with an ESI^+^ ion source. Full MS and MSMS were scanned in the mass range of 50–1250 *m*/*z*. UPLC-Q-TOF-MS data were captured using the DIA SWATH method. The collision energy was 35 V with a spread of 15 V.

### 2.8. LC-MS/MS Data Analysis

LC-MS data were analyzed and processed according to the procedure flow we previously reported [14,15]. In brief, the raw data files were imported into MS-Dial (version 4.7.0) for peak detection, MS2 deconvolution, metabolites identification, and peak alignment. The metabolites were identified by comparing MS1 and MS/MS with the MS-Dial public MSP database (VS15), whose items was washed and limited to those KEGG compounds with biological roles (br08001). In the compound annotation, the m/z tolerance was set with 2 mDa t for MS1 and 5 mDa for MS/MS data. Then, principal component analysis (PCA) and partial least squares discriminant analysis (PLS-DA) were performed and visualized using the Bioconductor package mixOmics in R language. The fold change (FC) was measured based on the normalized peak area. The *p*-value was calculated using the t test function in R. Volcano and Venn plots were plotted using the R package. Differential metabolites were filtered by a threshold of FC > 1.2 and *p* < 0.02. Metabolite pathway enrichment were performed using Metaboanalyst online tools (version 5.0) and the Bioconductor package FELLA (version 1.14) [16].

### 2.9. Molecular Docking

The conformer set of Grb1 was searched under the MMFF94S force field in Conflex 6.8.9. The most stable conformer of GRb1 was submitted to Sybyl-x 2.1 for molecular docking. The structures of target proteins SLC52A1 (PDB ID: 2BZE) and RFK (PDB ID: 1NB9) were retrieved from RCSB PDB database on March 10, 2022. However, the X-ray structures of SLC52A2 and SLC52A3 have not been reported yet. We thus acquired the 3D structures based on the human sequence (Q9HAB3 and Q9NQ40) of SLC52A2 and SLC52A3 from the AlphaFold Protein Structure Database (https://alphafold.ebi.ac.uk/, accessed on 10 March 2022), which provides 3D models generated by artificial intelligence. All of the proteins were prepared in Sybyl-x, including removing water and ligands, adding hydrogens and charges, and staged minimization. The binding site was generated using automatic mode in Sybyl-x. The docking was performed using Surflex-Dock GeomX module in Sybyl-x and allowed protein movement in docking. The interactions between GRb1 and the target proteins were visualized using LigPlot+ 2.2.4, based on the docking result with the highest total score.

### 2.10. Real-Time Quantitative PCR (RT-qPCR) Analysis

Control (G1), model (G2) and GRb1 treated group (G3) zebrafish larvae were cultured according to the protocol for metabolomic samples. Three replicates of 100 larvae per replicate were used for each group. Total RNA was extracted utilizing AG RNAex Pro reagent (Accurate biology, Changsha, China). cDNA was then synthesized by reverse transcription of RNA using Evo M-MLV (RT–kit with gDNA clean for qPCR II). and SYBR Green Pro Taq HS qPCR Kit (Accurate biology, Changsha, China) in a CFX 96 system (Bio-Rad Laboratories, Inc, Hercules, CA, USA) for cDNA quantification. Thermal parameters (40 cycles) were as follows: 95 °C for 5 s and 60 °C for 30 s. The primer sequences are listed in Table S1. β-actin was used as an internal reference gene. The fold change of the genes under test was assessed based on the 2^−ΔΔCT^ method.

### 2.11. ROS, Cell Death, MDA and CAT Assays

The zebrafish larvae (72 hpf) used in each assay experiment were divided into control, model and drug-treated groups. Each group had three biological replicates (10 larvae per replicate). The control group was incubated in E3 water. The model group was exposed to E3 water containing 4% glucose and 0.2 mM alloxan to induce a pathological diabetic state. The drug-treated groups were treated with more of the test drugs (riboflavin and GRb1) at different concentrations than the model group. Larvae from each group were cultured for 48 h and then used for ROS, cell death, MDA and CAT assays. 

#### 2.11.1. ROS Assay

The production of ROS in zebrafish larvae was detected using the fluorescent probe DCFH-DA. Zebrafish larvae were cultivated for 1 h at 28 °C in the dark after drug exposure by replacing the medium with fresh medium added to the fluorescent probe DCFH-DA, to a final concentration of 20 μg/mL, and other operations were similar to the assay described in Section 2.4.

#### 2.11.2. Cell Death Assay

Cell death in larvae was detected by AO staining. Dead cells show enhanced staining and brighter fluorescence. At the end of chemical exposure, the culture medium of larvae was replaced with 5 μg/mL of AO solution and hatched for 20 min at 28 °C, protected from light. Refer to Section 2.4 for the remaining operations.

#### 2.11.3. MDA Assay

At 120 hpf, 10 larvae from the same treatment were collected and homogenized with the extracts in the assay kit. The homogenates were centrifuged at 8000× *g* for 10 min at 4 °C and supernatants gathered for the assay. Protein concentration and MDA level were measured using the BCA Protein Concentration Assay Kit (Beyotime, Shanghai, China) and MDA content assay kit (Solarbio, Beijing, China), respectively.

#### 2.11.4. CAT Assay

At 120 hpf, 10 larvae from the same treatment were collected and homogenized with the extracts in the assay kit. The homogenates were centrifuged at 8000× *g* for 10 min at 4 °C and supernatants gathered for the assays. Protein concentration and CAT activity were measured using the BCA Protein Concentration Assay Kit (Beyotime, Shanghai, China) and CAT activity assay kit (Solarbio, Beijing, China), respectively.

### 2.12. Statistical Analysis

All bioassay data were analyzed on Prism 8. A *t*-test was used for the comparison of two groups, and one-way ANOVA was used for multi-component comparisons. All results are expressed as mean ± SEM. Different levels of significance are denoted by *p* < 0.05, *p* < 0.01 and *p* < 0.001.

## 3. Results

### 3.1. Bioguided Isolation

**Figure 2:**
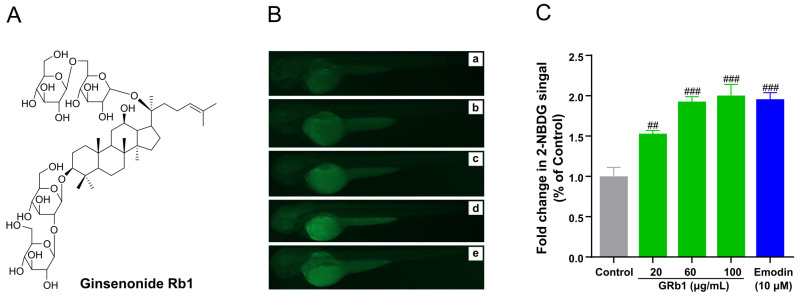
GRb1 induces 2-NBDG uptake in zebrafish larvae. (**A**) Chemical structures of ginsenoside Rb1. (**B**) Fluorescence microscopy images of zebrafish larvae treated with different concentrations of GRb1 (20, 60, 100 μg/mL) with 10 μM emodin as a positive control. Control (a), 20 μg/mL GRb1 (b), 60 μg/mL GRb1 (c), 100 μg/mL GRb1 (d) and 10 μM emodin (e). (**C**) Fold change in 2-NBDG absorption quantified by Image J. Results are expressed as mean ± SEM. ^###^ *p* < 0.001, ^##^ *p* < 0.01 vs. control.

Bioactivity guidance can lead directly to isolating the most promising functional molecules from a natural source. Herein, we use zebrafish larvae and 2-NBDG as a fluorescent indicator to mine the essential glucose-uptake promoter from American ginseng extract, as shown in Figure 1A. Zebrafish larvae are tiny enough to serve as a whole organism to indicate microgram-level samples sensitively and are well suited for targeted isolation for active components [17]. The higher intensity of fluorescence of the zebrafish indicated more effect of the component in promoting glucose uptake. As shown in Figure 1B,C, the extract EX1 (extracted by 100% EtOH) showed higher bioactivity than EX2 (extracted by 10% EtOH). In the chromatography column separation, the fraction Figure 1C showed better activity than the other fractions, which afforded the essential compound following the indication of 2-NBDG fluorescence in larvae. The component was identified as ginsenoside Rb1 (GRb1, Figure 2A) by comparing 1H, 13C NMR and HR-ESI MS data (Figures S1–S3) with reported data [18].

### 3.2. Glucose Uptake Promotion of GRb1

GRb1 has been reported to stimulate glucose uptake in 3T3-L1 cell lines [19]. Therefore, we explored the effect of GRb1 on metabolism in vivo from a metabolic perspective and its regulatory mechanisms that modulate glucose uptake. Before the exploration, we firstly reexamined the bioactivity on the whole organism zebrafish larvae model to guarantee the validity of metabolomic analysis. The natural product emodin was applied as a positive control, the essential component of the hypoglycemic herb rhubarb. Emodin is an anthraquinone compound of botanical origin that has been reported to promote glucose absorption and ameliorate diabetes in animal models but also causes diarrhea with long-term administration [20]. The fluorescence intensity of the larvae was significantly increased (*p* < 0.01) at a concentration of 20 μg/mL of GRb1 compared to the control group and in a dose-dependent manner (20, 60 and 100 μg/mL) (Figure 2B,C). This result verified that GRb1 could stimulate glucose transport in zebrafish.

### 3.3. Metabolomics Analysis

**Figure 3:**
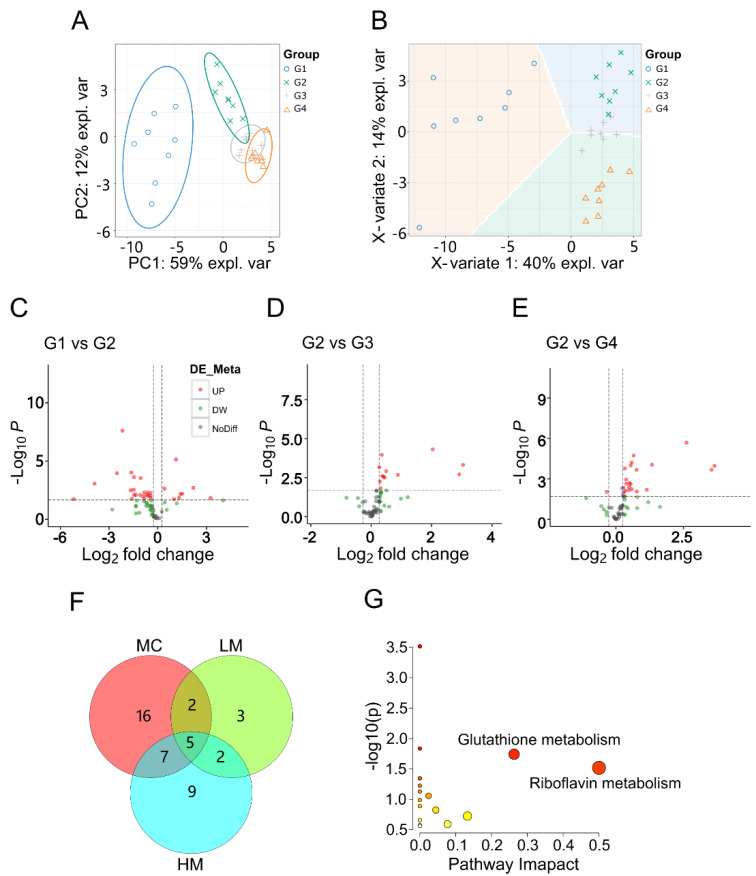
Metabolomic analysis of GRb1 treatment in diabetes zebrafish model. (**A**,**B**) Overall metabolic profiles of samples between control, model and test groups using PCA and PLS-DA plots. n = 8 per group. (**C**–**E**) Volcano plots of differential metabolites between G1 and G2, G2 and G3, and G2 and G4. G1, larvae exposed in E3 water; G2, larvae treated with 4% glucose; G3, larvae treated with 4% glucose + 100 μg/mL GRb1; G4, larvae treated with 4% glucose + 200 μg/mL GRb1. (**F**) Venn diagram of differential metabolite amounts. MC represents G2 vs. G1; LM represents G3 vs. G2; HM represents G4 vs. G2. (**G**) KEGG pathway maps for the differential metabolites.

We applied a differential metabolome strategy to explore metabolic changes after β-cell injury and GRb1 intervention, then located the major pivotal sites where GRb1 regulates metabolism in *vivo*. Metabolites from zebrafish larvae in the groups G1~G4 were detected on a UPLC-Q-TOF/MS in ESI positive ion mode and identified based on MS/MS fragments. G1 was the blank control larvae in E3 water, G2 larvae were treated with 4% glucose in E3 water, G3 larvae were exposed to 4% glucose and 100 μg/mL GRb1, and G4 larvae were exposed to 4% glucose and 200 μg/mL GRb1. Unsupervised PCA and supervised PLS-DA were used to assess the overall distribution and dispersion of samples in all groups (Figure 3A,B). Two principal components (PC) contributed 71% of the total variance in the PCA score plot (Figure 3A, each point represents a sample). The four groups of samples showed clear clustering by PCA component, indicating significant intergroup differences. The metabolite ellipses of the model group samples were significantly separated from those of the control group G1, which suggested a substantial change in the endogenous metabolites of the larvae under glucose treatment. In the direction of principal components, the low-dose group partially overlapped with the model group G1, but the high-dose group G4 showed a clear trend of separation from the model group G2.

In addition, PLS-DA plots (Figure 3B, each point represents one sample) were used to maximize the analysis of the intergroup differences, with the two PC accounting for 54% of the total variance. The results showed a clear separation and clustering among the four groups. Whole metabolites exploration indicated that GRb1 promoted the difference in metabolic profiles and suggested that GRb1 influenced the metabolic level of diabetes zebrafish. Notably, both the PCA and PLS-DA plots showed that the low-dose group (G3) was intermediate between the model (G2) and high-dose (G4) groups, suggesting that the overall changes in the metabolic group showed specific dose-dependent characteristics. These results illustrate that GRb1 supplementation alters the metabolic profile of diabetes larval metabolites.

### 3.4. Screening and Identification of Differential Metabolites

To filter out the differential metabolites, we set three comparative groups: MC (G2 vs. G1), LM (G3 vs. G2), and HM (G4 vs. G2). The criterion was set as fold change (FC) > 1.2 and statistical *p* < 0.02. The variation tendency was visualized in the volcano plots (Figure 3C–E). Each point in the volcano plots represents a metabolite, in red for clearly upregulated metabolites, green for clearly down-regulated metabolites, and gray for unchanged metabolites. The number of differential metabolites filtered out from the three comparative groups are depicted in Figure 3F. In the comparative group MC, 30 differential metabolites were screened out and related to the abnormal glucose metabolism induced by high glucose. In the comparative groups of LM and HM, 12 and 23 differential metabolites were filtered out. These differential metabolites were related to GRb1 intervention. Treatment with the high dose of GRb1 invoked more changeable metabolites than with the low dose. In the interaction of MC and LM and the interaction of the MC and HM, seven and 12 key metabolites were filtered out, which were considered to cover the metabolic regulatory targets. These key metabolites included amino acids, alkaloids, phenylpropanoids, phenols, sugars, flavonoids, nucleotides, lipids, and vitamins, as shown in Table S1. Both low and high doses of GRb1 interfered with the expression of five key differential metabolites, which were amino acids and phenylpropanoids, as listed in Table S2. In addition, seven metabolites can only be changed by high-dose GRb1, which are amino acids, nucleotides, lipids and vitamins.

### 3.5. Metabolic Pathway Analysis

**Figure 4:**
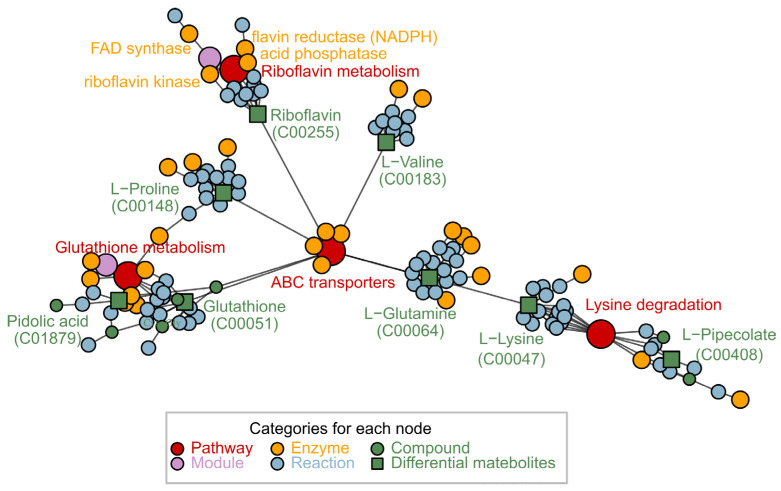
Network diagram related to differentially expressed metabolic pathways after GRb1 treatment by FELLA.

The apparent changes in the expression of differential metabolites across the comparison groups indicated that their metabolic pathways were significantly implicated. This may be due to the protective effect of GRb1 against diabetes in zebrafish larvae. To better understand how metabolite alterations are induced by GRb1 administration, we carried out pathway enrichment using the online toolkit Metaboanalyst 5.0 (accessed on 10 January 2022) to explore and visualize the metabolic pathways influenced by GRb1. Figure 3G shows the pathway enrichment results for 14 metabolites that were significantly changed after GRb1 (100 µg/mL and 200 µg/mL) treatment in diabetes zebrafish. As shown in Figure 3G, the size of the bubbles is positively correlated with the size of the pathway impact factor, and the color of the bubbles reflects *p*-value of the enrichment analysis. Darker colors represent smaller *p* values, meaning more significant enrichment. Six metabolic pathways were regulated after GRb1 treatment, including glutathione metabolism, riboflavin metabolism, purine metabolism, sphingolipid metabolism, alanine, aspartate and glutamate metabolism, arginine and proline metabolism. All the enrichment data, including *p*-values and impact values, are listed in Table 1. Our results indicated that GRb1 had a more significant effect on glutathione metabolism and riboflavin metabolism, especially riboflavin metabolism. In addition, we mapped the metabolic pathway network (Figure 4) using FELLA to facilitate a clearer view of the overall affected pathways and the corresponding vital metabolites.

### 3.6. Molecular Docking Validation to Riboflavin Pathway

**Figure 5:**
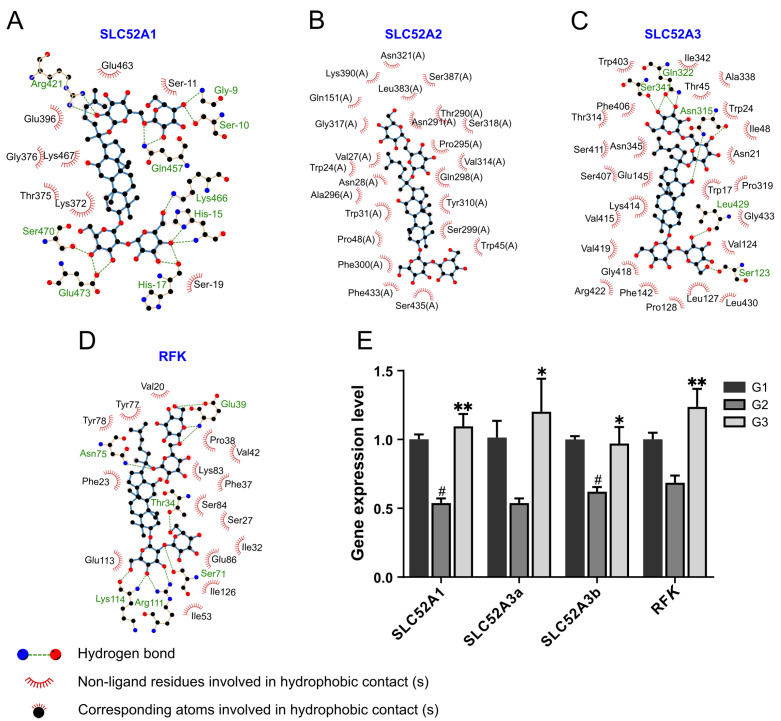
Verification of the role of GRb1 in riboflavin metabolic pathway. (**A**–**D**) Ligplot + results for schematic ligand interactions of 4 proteins related to riboflavin metabolic pathway with GRb1 showing hydrogen bonding and hydrophobic interactions. (**E**) Expression of the mRNA of the proteins related to riboflavin metabolic pathway in larvae after GRb1 treatment. Results are expressed as mean ± SEM, n = 3 per group. ^#^ *p* < 0.05 vs. G1. ** *p* < 0.01, * *p* < 0.05 vs. G2. G1, larvae exposed in E3 water; G2, larvae treated with 4% glucose; G3, larvae treated with 4% glucose + 100 μg/mL GRb1.

Given the metabolomic results showing riboflavin metabolism as the main pathway of GRb1 action in diabetes, we performed molecular docking analysis by Sybyl-x 2.1 to further evaluate the binding pattern of GRb1 with the upstream and downstream proteins SLC52A1, SLC52A2, SLC52A3, and RFK of riboflavin. GRb1 showed a high affinity with the four target proteins, with scores of 10.7, 12.5, 17.3 and 17.6 for SLC52A1, SLC52A2, SLC52A3 and RFK, respectively. The interactions between GRb1 and each protein were visualized using Ligplot+, as shown in Figure 5A–D. GRb1 was found to interact with nine amino acid residues of SLC52A1 (Arg421, Gly-9, Ser-10, Gln-457, Lys466, His-15, Ser470, Glu473 and His-17) via 14 hydrogen bonds. GRb1 had no hydrogen bonding interaction with SLC52A2. GRb1 had eight hydrogen bonds interaction with five amino acid residues (Ser341, Gln322, Asn315, Leu429 and Ser123) of SLC52A3. GRb1 interacted with six amino acid residues of RFK (Asn75, Thr34, Lys114, Arg111, Ser71 and Glu39) through 10 hydrogen bonds. GRb1 favorably interacted with specific amino acid residues of these four proteins through hydrophobic interactions. The above results revealed that GRb1 bound better to SLC52A1, SLC52A3 and RFK and less well to SLC52A2. It could be speculated that SLC52A1, SLC52A3 and RFK might be potent target proteins of GRb1.

### 3.7. RT-qPCR Verification to Riboflavin Metabolism

To further explore whether GRb1 affects the activity of riboflavin-related proteins, we investigated the effect of GRb1 on the expression modes of these proteins by quantitative real-time polymerase chain reaction (RT-qPCR). SLC52A3 has been reported to produce two transcript variants, SLC52A3a and SLC52A3b, during transcription, due to different transcriptional start sites [21]. SLC52A2 is one of the mammalian riboflavin transporters and is not present in zebrafish. We quantified the relative expression levels of SLC52A1, SLC52A3a, SLC52A3b and RFK mRNA in larvae (Figure 5E). The results showed that *SLC52A1* and *SLC52A3b* levels were significantly downregulated in the model group (G2) compared to the control group (G1), while GRb1 treatment (G3) reversed this downregulation. It is suggested that glucose intervention inhibited the expression of *SLC52A1*, *SLC52A3a*, *SLC52A3b* and *RFK*. On the contrary, GRb1 significantly upregulated the expression of *SLC52A1*, *SLC52A3a*, *SLC52A3b* and *RFK.*

### 3.8. Riboflavin Target Validation

**Figure 6:**
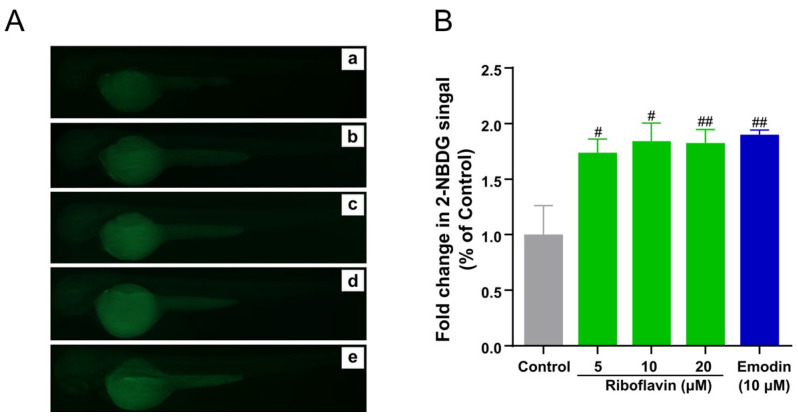
Riboflavin induces 2-NBDG uptake in zebrafish larvae. (**A**) Fluorescence microscopy images of zebrafish larvae treated with different concentrations of riboflavin (5, 10, 20 μM) with 10 μM emodin as a positive control. Control (a), 5 μM riboflavin (b), 10 μM riboflavin (c), 20 μM riboflavin (d) and 10 μM emodin (e). (**B**) Fold change in 2-NBDG absorption quantified by Image J. Results are expressed as mean ± SEM. ^##^ *p* < 0.01, ^#^ *p* < 0.05 vs. control.

Given our results suggesting that riboflavin is a significant target of GRb1, we further validated riboflavin regulation of glucose absorption. As shown in Figure 6, the uptake of 2-NBDG by larvae was significantly increased by treatment with 5~20 μM riboflavin compared with the control group. There was no significant difference in 2-NBDG uptake promotion between riboflavin and the positive control emodin. This demonstrated that riboflavin is a vital junction to promote glucose uptake.

### 3.9. GRb1 Ameliorates Oxidative Stress in Diabetes Zebrafish Model

**Figure 7:**
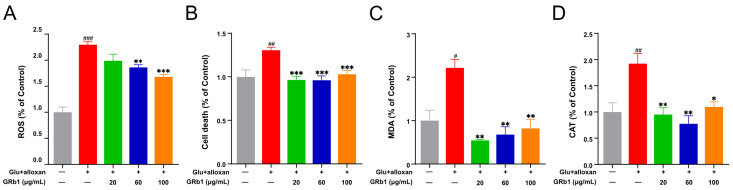
GRb1 treatment attenuates oxidative stress in a diabetes zebrafish model. ROS production (**A**), cell death (**B**), MDA levels (**C**) and CAT activity (**D**) in diabetes zebrafish larvae treated with different concentrations of GRb1. Results are expressed as mean ± SEM. ^###^ *p* < 0.001, ^##^ *p* < 0.01, ^#^ *p* < 0.05 vs. control. *** *p* < 0.001, ** *p* < 0.01, * *p* < 0.05 vs. model.

It is well known that riboflavin, the significant component of the flavoenzymes cofactor, is involved in redox reactions and energy metabolism in vivo, mainly through the respiratory chain in the form of FMN and FAD [22]. Riboflavin deficiency can cause oxidative stress [23]. Chronic hyperglycemia can induce oxidative stress and increase ROS in the body, resulting in oxidative damage to various cells, tissues, and organs [24]. Firstly, to determine whether GRb1 may regulate redox status in the diabetes model, we evaluated the influence of GRb1 treatment on ROS production, cell death, MDA levels, and CAT activity changes in diabetes larvae. As shown in Figure 7 and Figure S4, diabetes larvae showed a significant increase in ROS, cell death, MDA levels and CAT activity. GRb1 treatment significantly reduced ROS, cell death, MDA levels and CAT activity in diabetes larvae. These results demonstrated that GRb1 restored redox homeostasis in the diabetes larvae.

**Figure 8:**
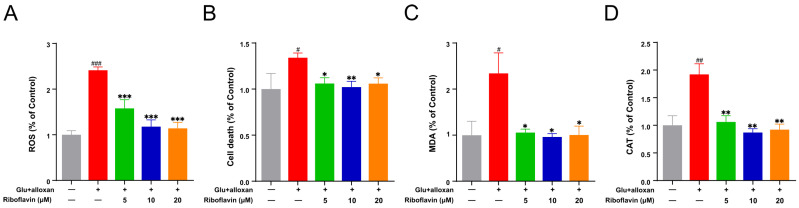
Riboflavin supplementation attenuates oxidative stress in a diabetes zebrafish model. ROS production ((**A**), cell death (**B**), MDA levels (**C**) and CAT activity (**D**) in diabetic zebrafish larvae treated with different concentrations of riboflavin. Results are expressed as mean ± SEM. ^###^ *p* < 0.001, ^##^ *p* < 0.01, ^#^ *p* < 0.05 vs. control. *** *p* < 0.001, ** *p* < 0.01, * *p* < 0.05 vs. model.

To further investigate the relationship between the GRb1 amelioration on oxidative stress and riboflavin effects, we also treated diabetes larvae with riboflavin and measured the oxidative stress levels of ROS, cell death, MDA and CAT activity in whole larval tissues. As shown in Figure 8 and Figure S5, ROS, cell death, MDA levels and CAT activity were significantly increased in diabetes larvae compared to the control group, while riboflavin intervention reversed their increases. The above observations demonstrate that GRb1 ameliorates diabetes by targeting riboflavin metabolism to regulate redox homeostasis.

## 4. Discussion

A sensitive and reliable phenotypic model can be used as an activity navigator to guide separation of the most potent active components from herbs. Zebrafish have proximate glucose metabolism to humans. The Fish glucose transporter protein genes *Glut2* and *Glut4* showed significant conservation with human homologs [25]. The function of the pancreas and liver, two essential organs involved in diabetes, are also significantly conserved between zebrafish and mammals [13]. In addition, the whole larva of zebrafish is transparent, which facilitates microscopic observation. Moreover, due to its rapid growth, it is possible to form appropriate disease models through early intervention.

Promoting the transport and absorption of glucose can accelerate glucose metabolism to stabilize the blood glucose level. The fluorescent probe 2-NBDG was generally used to visualize glucose uptake in zebrafish larvae [20], because 2-NBDG is a fluorescently labeled analog of 2-deoxyglucose that serves as glucose uptake and transport probe. Since the probe molecule has a higher binding capacity than the glucose transporter, it is harder to metabolize than glucose and more easily accumulates in skeletal muscle. Thus, the fluorescence intensity in zebrafish larvae with 2-NBDG can reflect the glucose uptake.

This study demonstrated that GRb1 is the most potent component in American ginseng that promotes glucose uptake. Although the activity of the compound GRb1 has been reported [26], its regulation on metabolism in diabetic vivo and the main targets that exert hypoglycemic effects are still unknown. GRb1 is one of the significant ginseng diol-type active ingredients in the genus Ginseng [27]. It can improve blood glucose and insulin resistance to prevent macrovascular/microvascular related complications by modulating oxidative stress, inflammatory response, autophagy and anti-apoptotic effects [28], in addition to promoting GLUT 4 translocation [29,30] and accelerating adipogenesis [31].

Diabetes mellitus is also a metabolic disease. The development of diabetes is accompanied by significant metabolic dysregulation. Therefore, we explored the metabolic regulation mechanism of GRb1 from the perspective of the metabolome. Our metabolomic analysis revealed that 30 endogenous metabolites of diabetic zebrafish larvae were disrupted and that GRb1 supplementation (100 μg/mL and 200 μg/mL) altered 14 metabolites. KEGG pathway enrichment based on the 14 metabolites showed they involved two main pathways, riboflavin metabolism and glutathione metabolism. The enriched pathways and related enzymes are shown in Figure 4. Since riboflavin metabolism was enriched with the highest pathway impact, and is a new finding of the GRb1 target, this pathway was selected for validation and regulation mechanism investigation.

Riboflavin is known as vitamin B2, which is generally not self-synthesized in animals and must be consumed in the diet [32]. Free riboflavin is absorbed and transported by the proteins SLC52A1, SLC52A2, SLC52A3 [33]. Then, the absorbed riboflavin is converted to flavin mononucleotide (FMN) by riboflavin kinase (RFK). These closely related proteins are potential targets of GRb1. We therefore performed molecular docking before proceeding with further validation. The two riboflavin transporter proteins SLC52A1 and SLC52A3 and RFK showed high affinities with GRb1 (Figure 5A–D). Their docking total scores were all higher than the general threshold of 12, especially the SLC52A3 and RFK that had scores higher than 17. In the high glucose-induced larvae, the gene expression of the transporters (SLC52A1, SLC52A3a, SLC52A3b) and RFK were suppressed. When the larvae were treated with GRb1, these gene expressions were restored back to normal. Thus, GRb1 accelerated the metabolism of riboflavin by upregulating the riboflavin transporters and metabolic enzyme RFK, which evoked the changes of riboflavin in different treatment groups.

Riboflavin and its downstream product FMN are involved in various redox reactions in vivo. Furthermore, our pathway enrichment also found that GRb1 regulation involved the glutathione metabolism pathway, which is also an essential pathway for oxidative stress. Glutathione (GSH) is an antioxidant that is catalyzed by glutathione peroxidase (GSH-Px) to glutathione disulfide (GSSG) and protects cells from oxidative stress. GSH/GSSG levels were measured to estimate oxidative stress status [34]. Oxidative stress is defined as an increased production or decreased scavenging of highly reactive molecules (i.e., reactive oxygen radicals and reactive nitrogen radicals). Oxidative stress is crucial in increasing risk factors for chronic diseases, especially diabetic vascular complications. Riboflavin supplementation has also been reported to improve oxidative stress in diabetes [35]. Oxidative stress causes the production of a large number of free radicals, which leads to lipid peroxidation and, consequently, cellular damage [36]. Superoxide dismutase (SOD) and CAT are the first stage of defense against free radicals by preventing their formation and proliferation [37]. MDA is a reactive aldehyde, which is a lipid peroxidation product, and MDA levels can indirectly reflect the extent of oxidative damage in cells [37]. Since oxidative stress was more pronounced in pathologically diabetic zebrafish (induced by alloxan combined with glucose), we investigated the antioxidant effects of GRb1 and riboflavin in pathologically diabetic zebrafish larvae by assessing the influence on ROS production, cell death, MDA levels and CAT activity. The results showed that both GRb1 and riboflavin significantly reversed the above four oxidative stress-related indicators in diabetes. These findings revealed that GRb1 could promote glucose consumption in diabetes by regulating riboflavin metabolism and redox homeostasis levels.

## 5. Conclusions

In summary, we acquired the most potential promotors (GRb1) of glucose uptake from a commonly used herbal medicine, American ginseng. UPLC-MS/MS based metabolomics as well as molecular docking combined with real-time quantitative PCR confirmed that GRb1 could bind riboflavin transporters SLC52A1 and SLC52A3 and RFK, affecting their expression levels. These results demonstrated that GRb1 restored redox balance, mainly by regulating riboflavin metabolism, promoting glucose uptake and alleviating hyperglycemia levels in vivo. Our findings disclosed the main hypoglycemic substances from American ginseng and elucidated its regulatory mechanism and targets. This could provide a basis for further developing hypoglycemic health products based on natural sources.

## Figures and Tables

**Table 1:** Pathway enrichment results for differential expressed metabolites (Impact > 0).

Pathway Name	Match Status	*p*	−log(*p*)	Impact
Glutathione metabolism	2/28	0.025	1.6017	0.26
Riboflavin metabolism	1/4	0.036	1.4469	0.50
Purine metabolism	2/66	0.117	0.93176	0.02
Sphingolipid metabolism	1/21	0.175	0.75733	0.04
Alanine, aspartate and glutamate metabolism	1/27	0.219	0.65886	0.13
Arginine and proline metabolism	1/38	0.295	0.52984	0.08

## Data Availability

Data are contained within the article and Supplementary Materials.

## Supplementary Materials

The following supporting information can be downloaded at: https://www.mdpi.com/article/10.3390/metabo12111011/s1, Figure S1: 1H NMR Spectrum of ginsenoside Rb1; Figure S2: 13C NMR Spectrum of ginsenoside Rb1; Figure S3: HR ESI MS spectrum of ginsenoside Rb1; Figure S4: Fluorescence microscopy images of ROS production (A) and cell death (B) in DM zebrafish larvae treated with different concentrations of GRb1; Figure S5: Fluorescence microscopy images of ROS production ((A) and cell death (B) in diabetic zebrafish larvae treated with different concentrations of riboflavin; Table S1: Primer sequences of the tested genes; Table S2: Differential expressed metabolites.
