# Supplementary material for: A New Mechanism for Ginsenoside Rb1 to Promote Glucose Uptake, Regulating Riboflavin Metabolism and Redox Homeostasis
Source: Metabolites. 2022 Oct 23;12(11):1011. doi: 10.3390/metabo12111011 (PMC9698532; doi:10.3390/metabo12111011)
Supplement: Supplementary file 1 [file metabolites-12-01011-s001.zip › metabolites-1963040-supplementary.pdf]

Supplementary materials

A New Mechanism for Ginsenoside Rb1 to Promote Glucose Uptake, Regulating Riboflavin Metabolism and Redox Homeostasis

Yihan Liu [1,†], Yuchan Deng [1,†], Fengyu Wang [1], Xiaoyi Liu [1], Jiaqi Wang [1], Jian Xiao [2], Cunli Zhang [1,*], and Qiang Zhang [1,2,*]

[1] Shaanxi Key Laboratory of Natural Products & Chemical Biology, College of Chemistry & Pharmacy, Northwest A&F University, Yangling 712100, China

[2] Shaanxi Key Laboratory of Phytochemistry, College of Chemistry and Chemical Engineering, Baoji University of Arts and Sciences, Baoji 721013, China

Contents

Abbreviation List 2

Figure S1. ^{1}H NMR Spectrum of Ginsenoside Rb1 3

Figure S2. ^{13}C NMR Spectrum of Ginsenoside Rb1 4

Figure S3. HR ESI MS spectrum of Ginsenoside Rb1 5

Figure S4. Fluorescence microscopy images of ROS production (A) and cell death (B) in DM Zebrafish larvae treated with different concentrations of GRb1 6

Figure S5. Fluorescence microscopy images of ROS production ((A) and cell death (B) in diabetic Zebrafish larvae treated with different concentrations of riboflavin. 7

Table S1. Primer sequences of the tested genes 9

Table S2. Differential expressed metabolites 9

Abbreviations List

2-NBDG, 2-Deoxy-2-[(7-nitro-2,1,3-benzoxadiazol-4-yl) amino]-D-glucose

AO, acridine orange

CAT, catalase

DCFH-DA, 2,7-Dichlorodihydrofluorescein diacetate

FAD, flavin adenine dinucleotide

FC, fold change

FMN, flavin mononucleotide

GRb1, ginsenoside Rb1

GSH, Glutathione

GSH-Px, glutathione peroxidase

GSSG, glutathione disulfide

hpf, hour post fertilization

IGT, impaired glucose tolerance

KEGG, Kyoto Encyclopedia of Genes and Genomes

MDA, malondialdehyde

PCA, principal component analysis

PLS-DA, partial least squares discriminant analysis

PTU, N-Phenylthiourea

RFK, riboflavin kinase

RT-qPCR, real-time quantitative

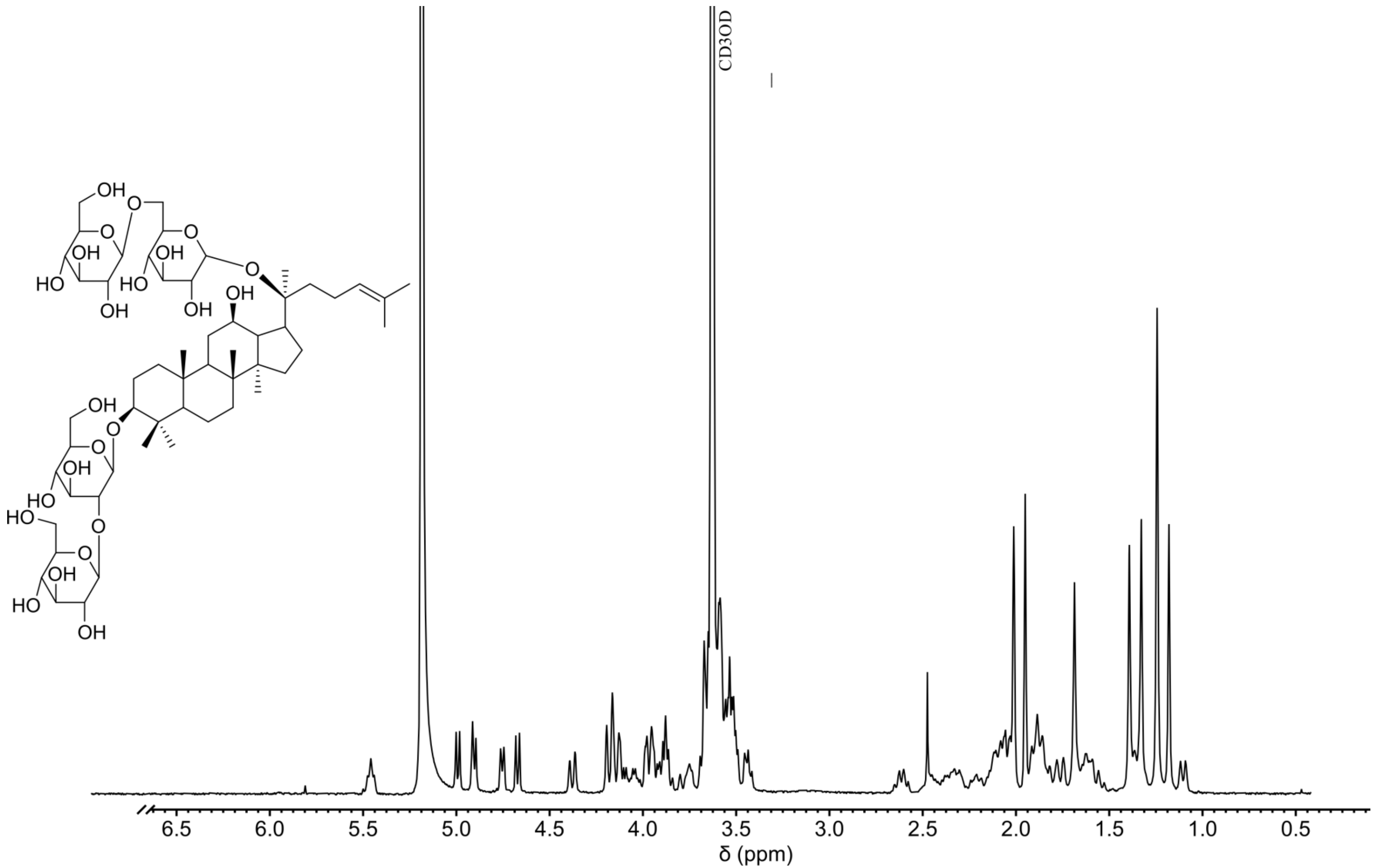

Figure S1. ^{1}H NMR Spectrum of Ginsenoside Rb1

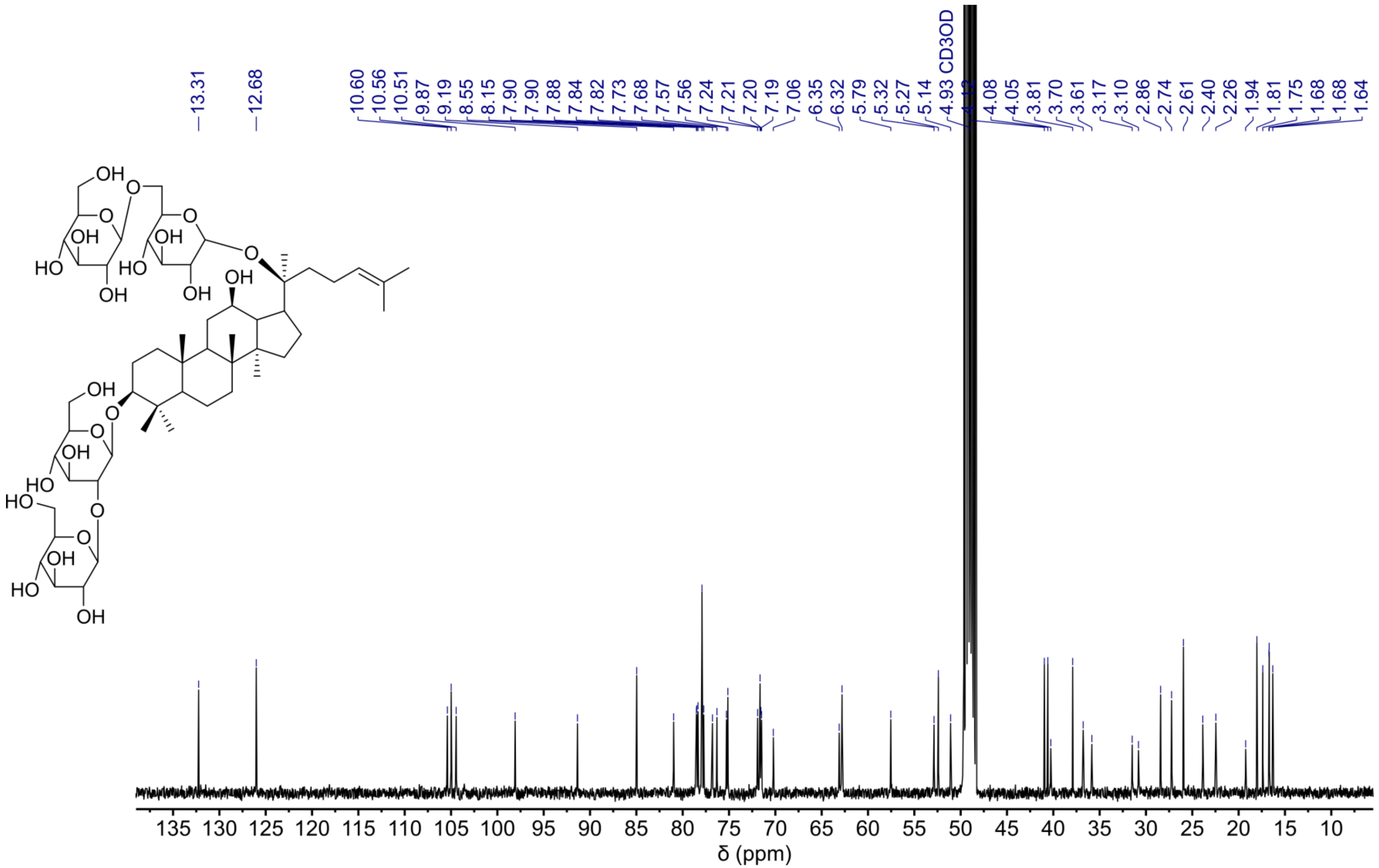

Figure S2. ^{13}C NMR Spectrum of Ginsenoside Rb1

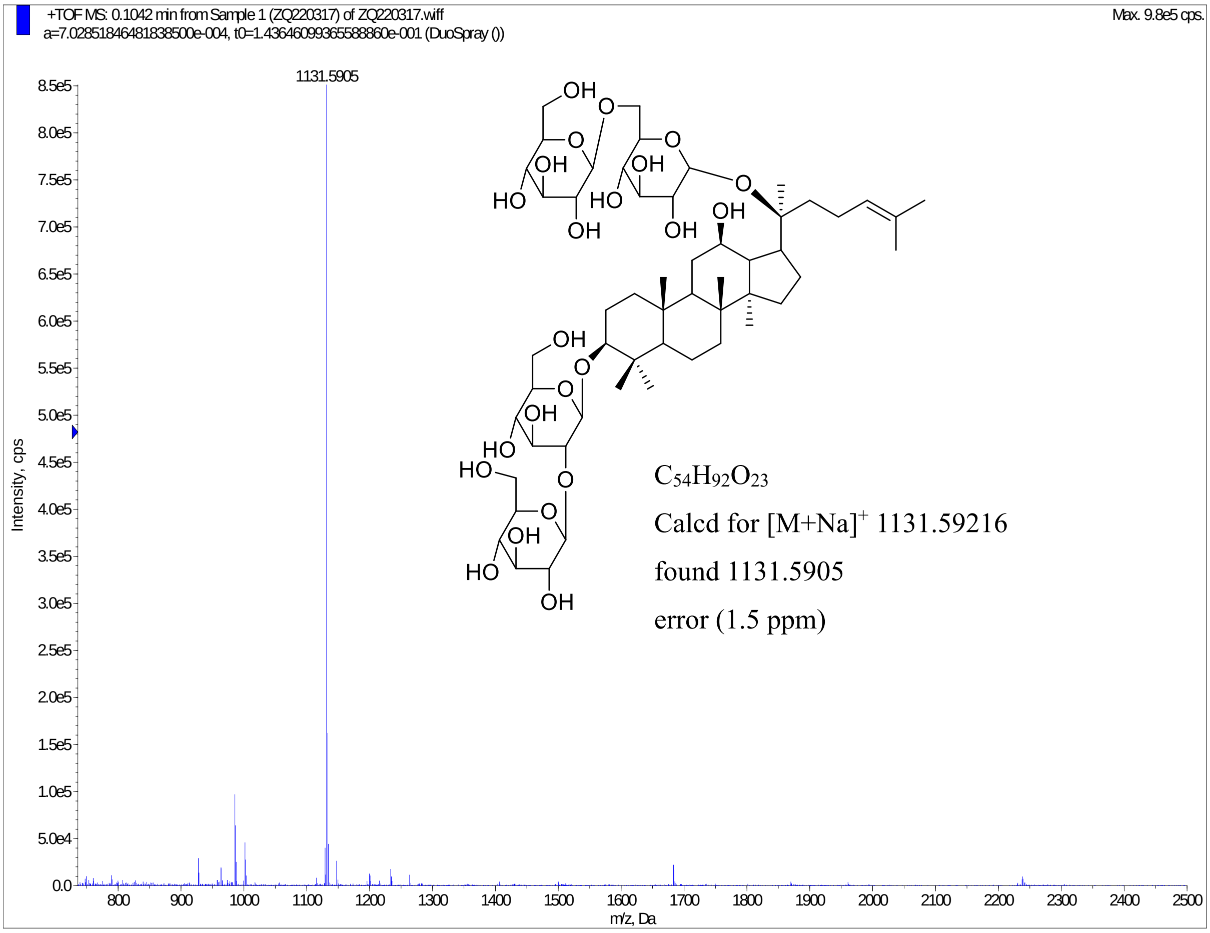

Figure S3. HR ESI MS spectrum of Ginsenoside Rb1

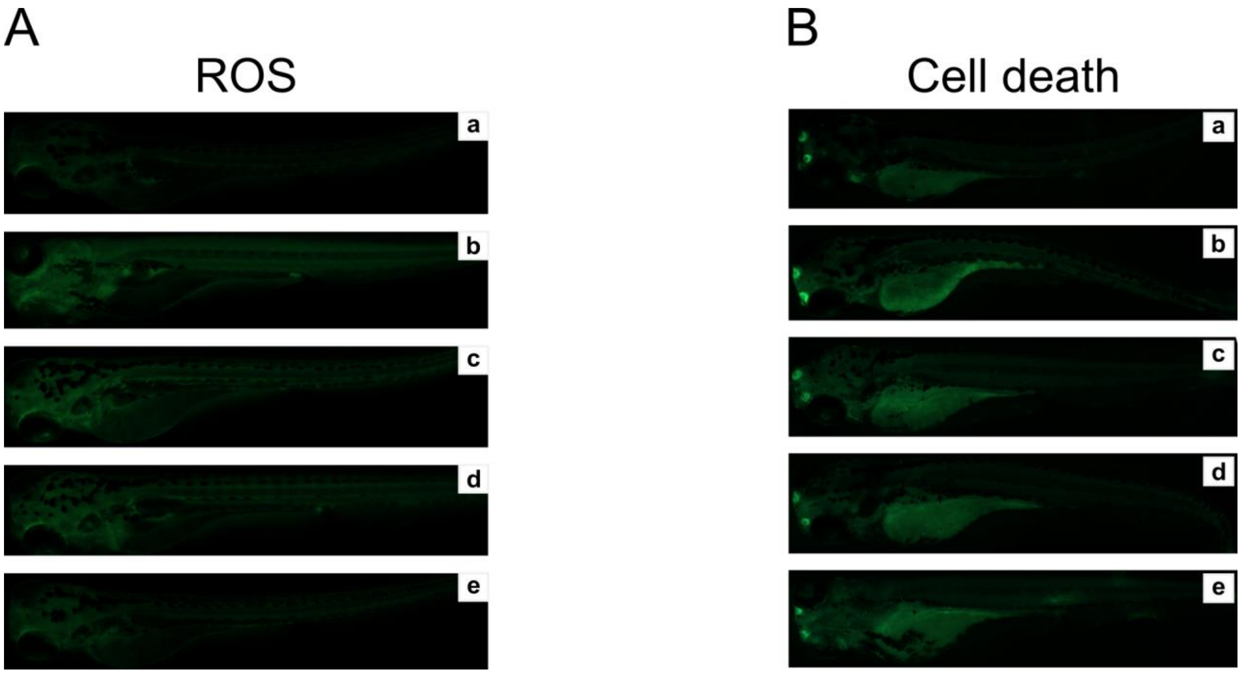

Figure S4. Fluorescence microscopy images of ROS production (A) and cell death (B) in DM Zebrafish larvae treated with different concentrations of GRb1. Control (a), model: 4% glucose + 0.2 mM alloxan (b), 20 μg/mL GRb1 (c), 60 μg/mL GRb1 (d), 100 μg/ mL GRb1 (e).

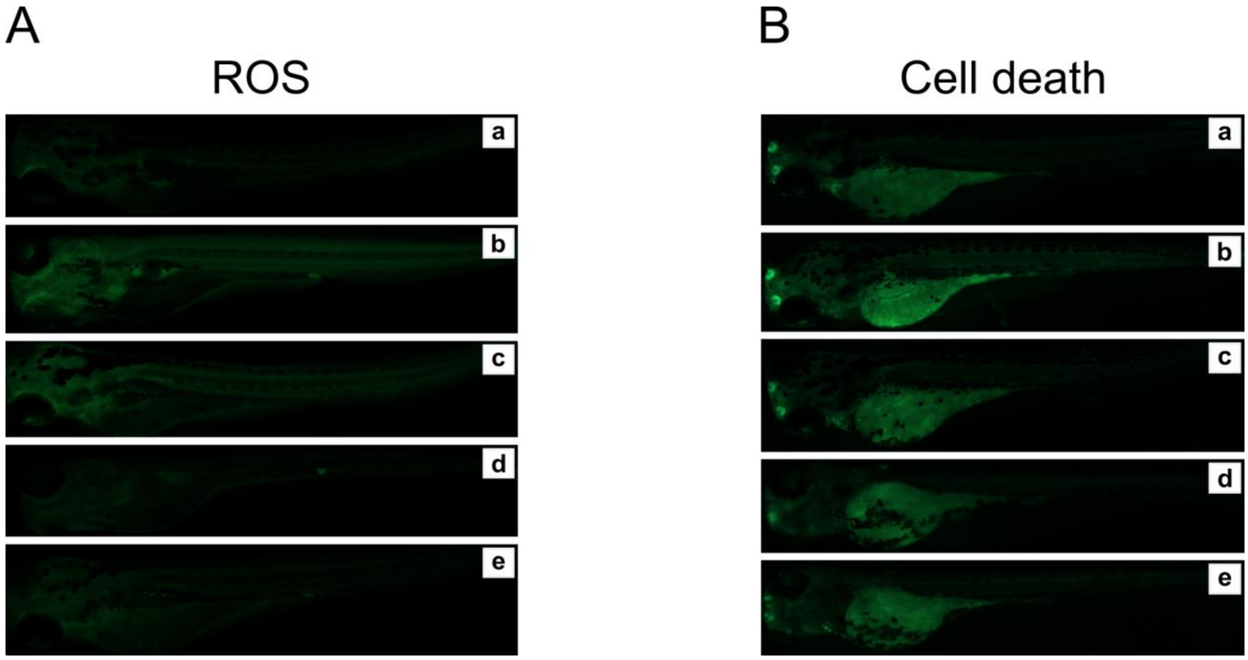

Figure S5. Fluorescence microscopy images of ROS production ((A) and cell death (B) in diabetic Zebrafish larvae treated with different concentrations of riboflavin. Control (a), model: 4% Glucose + 0.2 mM alloxan (b), 5 μM riboflavin (c), 10 μM riboflavin (d) and 20 μM riboflavin (e).

Table S1. Primer sequences of the tested genes

Gene	FP Sequence (5’-3’)	RP Sequence (5’-3’)
SLC52A1	GAACGCTTTTACGTTGCGGT	GACCTGTCTCCAGTTACGCC
SLC52A3a	ATCTTTGGACAACCTCGTAGCACAG	GGCAGGAAGGTAACAGACGAAGTG
SLC52A3b	AGCGCTGCATTACTGTCACT	CCAAACCGCTGAGACCTTCT
RFK	GCATCTACTACGGTTGGGCA	TTCAGGGCGAATGTAGCCAG
β-actin	F CGTGCTGTCTTCCCATCCA	TCACCAACGTAGCTGTCTTTCTG

Table S2. Differential expressed metabolites

KEGG ID	Metabolite name	Formula	RT/min	*m*/*z*	contrast
C00148	Proline	$C_5H_9NO_2$	1.035	116.0693	G3 *vs*. G2, G4 *vs*. G2
C00408	L-Pipecolate	$C_6H_{11}NO_2$	0.952	130.0853	G3 *vs*. G2, G4 *vs*. G2
C00047	Lysine	$C_6H_{14}N_2O_2$	0.948	147.1122	G3 *vs*. G2, G4 *vs*. G2
C09315	Umbelliferone	$C_9H_6O_3$	6.988	163.0384	G3 *vs*. G2, G4 *vs*. G2
C01742	Palatinose	$C_{12}H_{22}O_{11}$	6.520	360.1505	G3 *vs*. G2, G4 *vs*. G2
C10453	Eugenol	$C_{10}H_{12}O_2$	4.932	165.0895	G3 *vs*. G2
C17957	Irigenin	$C_{18}H_{16}O_8$	5.263	361.0858	G3 *vs*. G2
C00183	Valine	$C_5H_{11}NO_2$	1.028	118.0853	G4 *vs*. G2
C01879	Pidolic acid	$C_5H_7NO_3$	1.030	130.0490	G4 *vs*. G2
C00262	Hypoxanthine	$C_5H_4N_4O$	1.418	137.0447	G4 *vs*. G2
C00064	Glutamine	$C_5H_{10}N_2O_3$	1.033	147.0765	G4 *vs*. G2
C00319	Sphing-4-enine	$C_{18}H_{37}NO_2$	12.021	300.2901	G4 *vs*. G2
C00051	Glutathione	$C_{10}H_{17}N_3O_6S$	1.081	308.0891	G4 *vs*. G2
C00255	Riboflavin	$C_{17}H_{20}N_4O_6$	3.184	377.1455	G4 *vs*. G2
